# Ascites due to right atrial myxoma in a haemodialysis patient

**DOI:** 10.1186/1471-2369-7-4

**Published:** 2006-03-06

**Authors:** Anindya Banerjee, Andrew Davenport

**Affiliations:** 1 University College London, Centre for Nephrology, Royal Free Hospital, London, UK

## Abstract

**Background:**

Persistent fluid overload in patients on renal replacement therapy despite good dialysis adequacy or obvious cardiac dysfunction should prompt a search for rarer causes.

**Case presentation:**

We report here a rare cause of persistent peripheral oedema and ascites in a well-dialysed patient. CT scanning revealed a right atrial myxoma that was later confirmed on an echocardiogram.

**Conclusion:**

Fluid overload states are common in patients on dialysis. Common causes are inadequacy of dialysis and non-compliance. Where aetiology is not easily apparent further investigations into rarer causes should be sought.

## Background

Cardiovascular disease is responsible for about 50% of deaths in patients with end stage renal disease on dialysis. Such patients often show evidence of fluid overload. This is usually attributable to poor fluid removal, often associated with inadequate dialysis, and/or congestive cardiac failure. This can often be managed by a period of intensive dialysis with appropriate ultrafiltration [1]. Where a cause for fluid overload is not readily identifiable however, and fails to respond to increased dialysis, it is important to look for other contributory factors. We report here a case of right atrial myxoma in a patient on haemodialysis who presented with features of congestive cardiac failure, in particular, ascites that failed to respond to increased dialysis therapy.

## Case report

A 75 year old patient with end stage renal failure secondary to diabetes and hypertension who had been regularly dialyzed for 11 years presented with several weeks history of reduced appetite, weight loss, tiredness and nausea. He had also noted abdominal distension, and more recently ankle swelling.

On examination he was apyrexial, blood pressure 135/69 mmHg, an indeterminate jugular venous pressure (due to numerous previous central venous dialysis catheters), normal heart and chest sounds. He had signs of congestive cardiac failure with bilateral oedema to the mid calf and a 3 cm smooth hepatomegaly with moderate ascites.

Blood tests showed a haemoglobin of 13.8 g/dl, an albumin of 36 g/dl, elevated alkaline phosphatase of 418 iu/L (normal < 120 iu/l), gamma glutamyl-transferase 383 u/l (normal < 55 u/l) and C reactive protein of 62 mg/L (normal < 5 mg/l). Transaminases were normal with aspartate transaminase of 43 u/l and alanine transaminase 32 (normal < 45 u/l). Hepatitis B core antibody had been positive for many years.

He was well dialysed through a transposed native brachio-basilic fistula with a urea reduction ratio of 70% and Kt/v > 1.2. We attempted to reduce his dry weight over a few dialysis sessions without any success in reducing ascites.

The persistent ascites prompted further tests. Ascitic fluid tap revealed a sterile transudate with protein concentration of 29 g/dl (serum albumin 34 g/dl). Abdominal ultrasound scan showed moderate ascites with normal liver architecture without biliary dilatation or parenchymal lesions.

**Figure 1:**
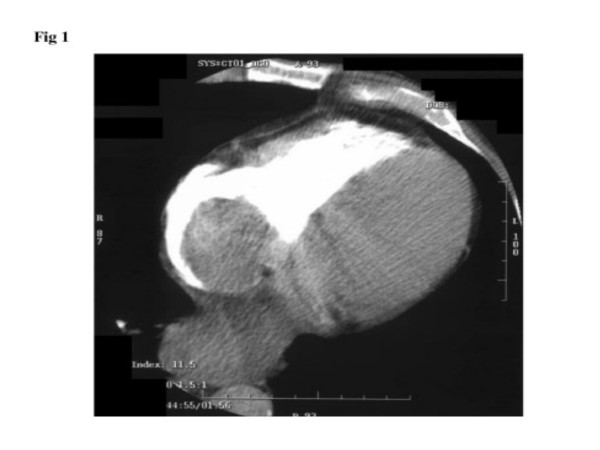
CT scan showing soft tissue mass within atrium which is isodense with myocardium and appearing to arise from the atrial wall by a stalk.

A diagnostic computed tomogram of the chest was obtained (fig 1). This showed a soft tissue mass, 5 cm × 4 cm within the right atrium which was confirmed on a repeat echocardiogram to be a right atrial myxoma. There was no evidence of a mass from an earlier echocardiogram in 2002, which reported an ejection fraction of 63% with mild tricuspid regurgitation and moderate left ventricular hypertrophy.

Although open-heart surgery was planned, he had a sudden cardiac death from an electrical-mechanical dissociation with no detectable cardiac output, probably related to occlusion of the tricuspid valve.

## Discussion

Right atrial myxomas constitute 25% of the exceedingly rare group of patients with primary tumours of the heart (incidence 0.001–0.33%) [2,3]. Symptoms vary from constitutional to thrombo-embolic and obstructive, the most common being congestive cardiac failure [4]. de novo primary cardiac tumours in patients on chronic haemodialysis as in our case however are rare and have not been reported before. There are reports though of right atrial masses from organised clots or thrombus in longstanding haemodialysis patients dialysing via internal jugular semi-permanent catheters [5]. Persistent ascites that does not respond to aggressive fluid removal by daily haemodialysis and ultrafiltration [1] could be a clue to such rarer causal factors.

Pericardial effusion could be another cause of fluid retention with ascites particularly in an inadequately dialysed patient, but also following an intercurrent illness or as part of a connective tissue disease in a well-dialysed patient. Uraemic pericarditis describes pericarditis before renal replacement therapy or within 8 weeks of initiation, whereas dialysis pericarditis occurs usually after ≥ 8 weeks of dialysis. The reported incidence of dialysis pericarditis ranges from 2 to 21% [6].

Our patient had been well dialyzed prior to admission and did not respond to a planned reduction in dry weight. He did have a generalized inflammatory state, characterized by weight loss and elevated C-reactive protein. Constitutional features such as fever and weight loss are related to cytokines, including interleukin-6 released from atrial myxomas [7].

Transthoracic echocardiography is usually the initial diagnostic test in patients with a suspected cardiac mass. However, its small field of views and insufficient acoustic window in some patients restricts this technique. Magnetic resonance imaging (MRI) and multislice spiral computed tomography allow for detailed delineation of intra and pericardiac tumors, their extent, and their influence on cardiac function [8]. Although an echocardiogram and a CT scan were requested for our patient the CT was available earlier and the findings were corroborated on a later echocardiogram.

## Conclusion

Features of nephrogenic ascites or congestive cardiac failure in a patient on renal replacement treatment who is adequately dialysed, and in whom the fluid overload state does not respond to intensification of dialysis and fluid removal including daily dialysis, should prompt further search for another underlying aetiology, as treatment options may vary.

## Competing interests

The author(s) declare that they have no competing interests.

## Authors' contributions

Both authors, AB and AD have contributed equally in the conception and design of this case report, in drafting the manuscript and revising it.

## Pre-publication history

The pre-publication history for this paper can be accessed here:


